# Acute Hydrocephalus Following a Spontaneous Ventriculoperitoneal Shunt Catheter Fracture With Scrotal Migration

**DOI:** 10.7759/cureus.14554

**Published:** 2021-04-19

**Authors:** Caio Perret, Raphael Bertani, Barbara Pilon, Stefan W Koester, Hugo C Schiavini

**Affiliations:** 1 Neurosurgery, Hospital Municipal Miguel Couto, Rio de Janeiro, BRA; 2 Neurosurgery, Vanderbilt University School of Medicine, Nashville, USA

**Keywords:** ventriculoperitoneal shunt, surgery complication, scrotum, surgial complications, hydrocephalus

## Abstract

Genitalia-related complications of ventriculoperitoneal shunts, such as scrotal migrations, are rare and most frequently presenting during the first year of the system placement, usually in the pediatric population, due to several factors, including vaginal process patency and increased abdominal pressure. Despite being typically benign, hernias, hydroceles, perforations, and catheter migration to the scrotum can lead to permanent disabilities and lethal complications, such as ventriculoperitoneal shunt dysfunction. We report a case of a late-onset, atraumatic, ventriculoperitoneal shunt fracture and catheter migration to the scrotum in a 22-year-old male, six years after its implantation, presenting in the emergency department due to acute hydrocephalus symptoms.

## Introduction

The vast majority of complications involving ventriculoperitoneal (VP) shunt procedures are presented at an average of three to four months of surgery, mainly in the pediatric population and somehow related to the distal catheter functioning [1,2]. Although distal catheter migration usually produces mild and benign implications, it might result in VP shunt dysfunction (and ultimately acute hydrocephalus as reported), testicle torsion, or perforations, which are disabling and possibly lethal conditions [1-5]. The distal catheter's scrotal migration is a poorly characterized, rare condition with no consensus regarding management due to its irregular and variable presentation [3]. To date, there are less than 30 cases reported involving distal catheter migration to the scrotum. To the best of our knowledge, none of them involved non-traumatic system fractures and distal catheter migration.

Henceforth, the elucidation of the underlying mechanisms involving distal catheter migration is still unclear in medical literature and might be aided by the reports of series and cases with variable presentations of this condition. This study reports the first case of a young adult patient that presented a scrotal migration of non-traumatic distal catheter fracture, with no inguinal hernia history upon admission.

## Case presentation

**Figure 1:**
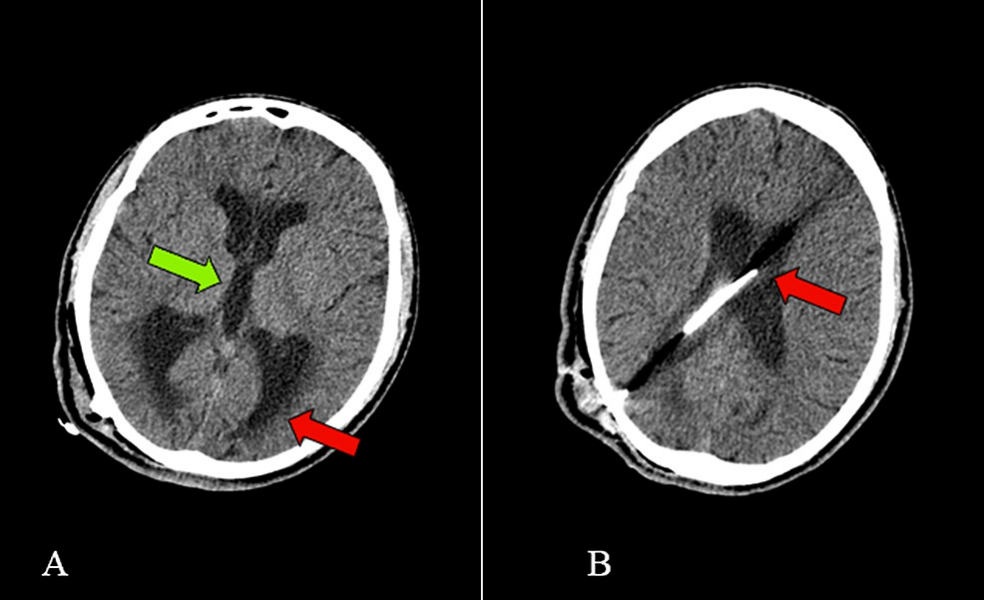
Hydrocephalus and proximal catheter (A) Axial section of a CT scan showing dilated lateral ventricles (red arrow) and dilated third ventricle (green arrow). (B) Axial section of a CT scan showing the position of proximal catheter (red arrow).

**Figure 2:**
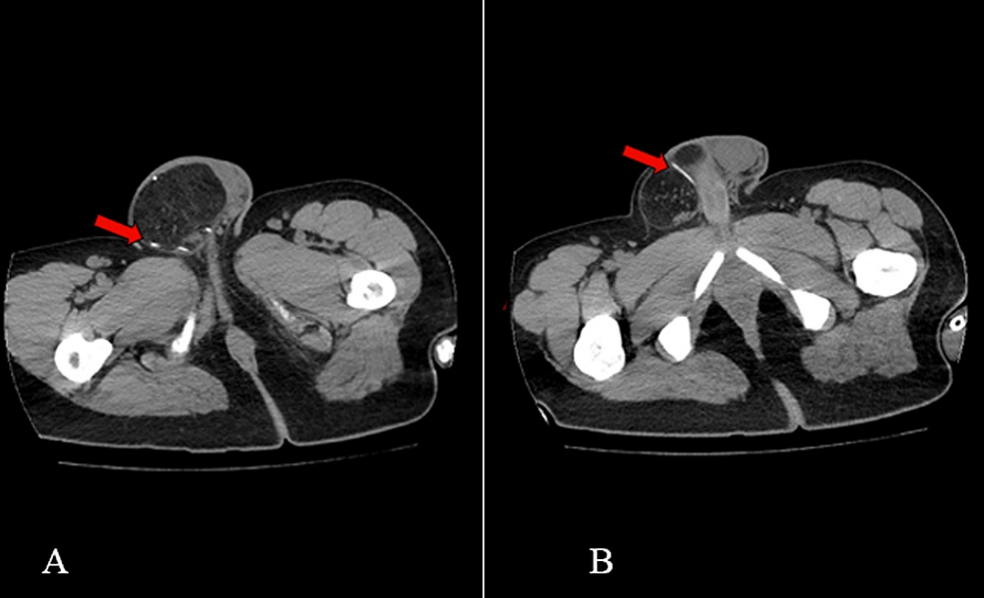
Migration of abdominal catheter Axial sections of abdominal CT scan showing hyperdensity (red arrows) corresponding to a coiled catheter (A) and a straight catheter (B) inside the scrotum, having migrated with the inguinal hernia.

A 22-year-old male was presented to the emergency department with a seven-day medical history of headache, reduced consciousness level, and vomiting. The patient was admitted with a Glasgow Coma Scale score of 13 (E:3 V: 4 M:6), photoreactive pupils, and no other significant deficits. The family companion referred to a unilateral scrotal protruding and painful mass noticed by the patient two days following the first symptom. A slow filling of the occipital ventriculoperitoneal shunt valve was detected during physical examination. The subject was otherwise healthy. Admission head CT scan showed hydrocephalus and an appropriate proximal catheter positioning (Figure 1). Distal CT scan tridimensional reconstruction showed catheter discontinuation around the thoracoabdominal transition level (Figure 2). Further evaluation showed a hypodense collection in the scrotal region with an intrinsic filamentary coiled hyperdensity within the mass, suggesting the catheter placement within the scrotum.

General surgery assessment performed on the same anesthetic procedure suggested an indirect inguinal hernia containing a peritoneal fluid-filled fold, with no intestinal loop involved. The patient was submitted to a VP shunt revision surgery and further open inguinal hernia surgical repairment. He was discharged on day 2 of in-hospital admission with no complaints or other complications. The patient authorized all the information and images provided in this report through an official personal statement in agreement with our ethics committee.

## Discussion

Until this article's literature research, there was a total of 29 case reports involving scrotal complications of VP shunting procedures. A case series on this condition involving 25 patients has reported a 6.8 months interval between surgery and symptom onset [5], in contrast with another study, published in 2017, reporting a three-to four-month interval. In contrast, our patient presented with an atraumatic fracture of the distal catheter leading to its migration at eight years following the procedure. In consensus with other reports, shunt migration was more common on the right side, as in the presented case, which is partially explained by the incidence of hernias in the pediatric age group [4].

Despite the scarcity of information regarding management, etiology, and risk factors of the distal catheter migration to the scrotum, the literature reveals an incidence of inguinal hernias following shunting as high as 16.8% in the pediatric population, and mostly being the first clinical repercussion of a patent vaginal process, present in up to 90% of male newborns and 15% of male adults [3-5].

Although distal catheter migration usually produces mild and benign implications, it might result in VP shunt dysfunction (and ultimately acute hydrocephalus as reported) and acute scrotum/testicle torsion perforations, which are disabling and possibly lethal conditions. Multifactorial mechanisms underlying the condition and the risk factors involved hypothetical but somehow consensual among the available literature.

There are several hypotheses around the mechanism and predisposing factors of scrotal migration. The most widely accepted theory is based on the fact that shunting leads to increased intra-abdominal pressure due to the drainage of cerebral-spinal fluid into the abdominal cavity, resulting in dissection and re-opening (or prevention of the closure) of the vaginal process, known to be the leading cause of inguinal hernias in the pediatric population. Furthermore, peritoneal cavities volume is known to be related to the body surface, in the proportion of 80mL/m^2 ^[6]. A small peritoneal cavity, such as present in children, might predispose this downward migration of the distal catheter in increased abdominal pressure situations. In this specific case, the patient presented an asymptomatic patent vaginal process seen in the surgical correction.

Considering the scarce information on this rare condition and the lack of guidelines in these situations, the VP shunt revision followed by surgical closure of the patent vaginal process seems consensual in the reports and series available in the literature [7,8]. Our patient was submitted to a VP shunt revision, followed by inguinal and abdominal cavity exploration for the patent vaginal process closure in the same anesthetic period. As the proximal catheter and valve function and patency could be proven during the revision surgery, only the distal catheter was changed and connected to the system.

Despite the absence of proven modifiable factors involving this condition in the literature, some deductible factors are hypothesized to be related to distal catheter migration when considering non-fractured systems. Low, fixed-pressure valves and the extra-lengthy catheters employed to avoid reoperation (especially when considering growing individuals) are the more widely accepted factors involved. Conversely, this report presents the first documented case of a distal catheter fracture with a scrotal migration to the best of our knowledge, resulting in a non-functioning VP shunt. Some of the factors potentially related to the atraumatic system fracture are the first surgery age onset, taken during the individual growth spurt, and the extra-tight peritoneal-end sutures, resulting in a traction pressure of the distal catheter [3].

Our experience proposes the loose suture sealing of the abdominal catheter to avoid excessive traction of the distal catheter, especially in individuals expected to present a significant stature increasing in the future. Another essential option to consider in these situations, when available, is the laparoscopic placement of the distal catheter in the subdiaphragmatic space, which is associated with a significant reduction of valve dysfunction (including fractures) [9].

## Conclusions

This article reports a rare presentation of VP shunt dysfunction being the first to describe an acute hydrocephalus presentation following non-traumatic distal catheter fracture and scrotal migration. Even though distal catheter migration is not an avoidable complication, some surgical strategies are hypothesized to reduce its incidence, as reported in this study. Increasing awareness of such complications may provide means to avoid them and provide better care to the patients.
